# Effectiveness and feasibility of smoking counselling: a randomized controlled trial in an Italian emergency department

**DOI:** 10.1093/eurpub/ckab114

**Published:** 2021-07-12

**Authors:** Luigi Mario Castello, Chiara Airoldi, Marco Baldrighi, Sara Bortoluzzi, Liborio Martino Cammarata, Livia Franchetti Pardo, Clara Ada Gardino, Anil Babu Payedimarri, Matteo Giorchino, Giovanni Pistone, Viviana Stampini, Gian Carlo Avanzi, Fabrizio Faggiano

**Affiliations:** 1 Department of Translational Medicine, Università degli Studi del Piemonte Orientale, Novara, Italy; 2 Emergency Department, “Maggiore della Carità” University Hospital, Novara, Italy; 3 Centro per il Trattamento del Tabagismo, Local Health Unit, Novara, Italy

## Abstract

**Background:**

5A’s counselling is recommended for screening and treating patients with smoking addiction. The emergency department (ED) setting might be a suitable environment for conducting interventions for smoking cessation. The present study aims to determine the feasibility and effectiveness on smoking cessation of 5A’s counselling administered to ED patients by nurses.

**Methods:**

Parallel group randomized trial assessing 5A’s counselling for smoking cessation vs. usual care at a University Hospital in the North of Italy. The primary end-point was prevalence of tobacco-free patients. The secondary outcomes at 6- and 12-month follow-up were (i) consecutive past 30-day smoking abstinence; (ii) past 7-day 50%, or more, decrease in daily tobacco consumption over baseline; and (iii) number of attempts to quit smoking.

**Results:**

A total of 480 patients were randomized to intervention (*n* = 262) or usual care (*n* = 218). Intention to treat analysis displayed no differences in primary and secondary outcomes between groups. A slight but not statistically significant enhancement in cessation was recorded in the intervention group [relative risk (RR) = 1.04, 95% confidence interval (CI) = 0.58–1.87] at 6 months, whereas a reversed observation at 12 months (RR = 0.86, 95% CI = 0.50–1.47). Similar results were obtained for the secondary outcomes. Per protocol analysis increased the size of the results. Of the 126 smokers receiving counselling, 18 were visited and treated at the local smoking cessation centre, with 12 of them successfully completing the treatment.

**Conclusion:**

The results of this study indicate that the ED is not a suited environment for 5A’s counselling.

## Introduction

Smoking is a major public health issue worldwide. This is particularly worrisome in Italy, where 25.7% of adults (18–69 years old) currently smoke tobacco.^1^

Moderate-quality evidence suggests that brief intervention performed by nurses to promote and support smoking cessation can increase the number of people achieving prolonged abstinence.^2^

The five major components of 5A’s smoking cessation intervention in the healthcare setting are the following: (i) ask every patient about tobacco use; (ii) advice current smokers to quit; (iii) assess the patient’s motivation to quit; (iv) assist both patients who want to quit and patients who are not interested in quitting using motivational interventions; and (v) arrange the follow-up schedule.^3^ The availability of a protocol for brief effective interventions can be extremely useful to a busy clinical team willing to conduct smoking cessation counselling on the ward.

Among emergency department (ED) patients, there is a higher prevalence of smokers compared to the general population (21–48%).^4^ Furthermore, each ED access usually represents a ‘teachable moment’, that is a particular situation when an illness directly related to the patient’s lifestyle behaviour makes him/her aware of the potential benefits that may result from changing such behaviour.^5^ The ‘teachable moment’ is therefore characterized by a strong and unique relation between the patient and the healthcare professional.^6^ In this regard, the Society for the Academic Emergency Medicine and Public Health and Education Task Force Preventive Services Work Group recommends to administer smoking cessation counselling to all smokers admitted to the ED.^7^ However, the implementation of counselling programs is often hindered by constant ED crowding and healthcare professionals’ discomfort worldwide.^8^ Indeed, 57.2% of US emergency physicians, when interviewed about this recommendation, expressed concern about the appropriateness of smoking cessation counselling intervention in the emergency clinical setting.^9^

In the last two decades, several large-N studies investigating the feasibility and efficacy of counselling for smoking cessation during ED stay have failed to demonstrate a statistically significant increase in smoking quit rates in patients receiving counselling at 3-,^10–13^ 6-^12^ and 12-month follow-up.^14^ Nonetheless, the results of these studies generally lean towards a better outcome, albeit not statistically significant, in smokers who receive the intervention during the ED stay.

Although a systematic review has recently shown a stronger impact of smoking cessation interventions conducted in EDs compared to other healthcare settings, it did not discriminate the role of specific counselling from that of the health visit itself.^4^ In order to fill this gap and assess the sustainability of smoking cessation intervention in a European context, here we have conducted a randomized clinical trial (RCT) aimed to evaluate the feasibility and effectiveness of 5A’s counselling for smoking cessation performed by nurses in the ED setting of a medium-sized city in northern Italy.

## Methods

### Study design

The study was a parallel group RCT with an allocation ratio of 1:1. The study was entirely conducted at the ED at ‘Maggiore della Carità’ University Hospital, Novara, Italy. Eligible patients were randomly assigned to intervention (i.e. 5A’s counselling) or control arm (i.e. usual care). The patients’ smoking habits were assessed by means of an *ad hoc* self-administered questionnaire at baseline. The protocol registration number is: NCT04107753.

The study was approved by the Ethical Committee of the University Hospital of Novara on 7 January 2016 (n. 602/CE).

### Population

The study population included individuals aged 18 years old or older admitted to the ED from April 2017 to May 2018, self-declared as ‘current smokers’, who gave their written informed consent. Exclusion criteria were (i) an emergency condition labelled with a red priority code according to the Italian triage protocol; (ii) assignment of the patient to a nurse not previously trained in 5A’s counselling; (iii) a mental illness or a history of psychiatric illness; (iv) inability to understand either written or oral Italian; and (v) ongoing treatment for tobacco addiction.

### Sample size

For sample size calculation, we assumed a cessation rate in the general population of 8.4%,^15^ with a 1.6 relative risk (RR) for the 12-month point-estimated tobacco abstinence in the intervention vs. usual care group, based on Cochrane systematic reviews.^2^^,^^15–18^ With this assumptions, a sample size of 1200 patients—600 for each arm of the study—was needed to obtain a desired two-tailed significance level of 95% (type I error = 0.05) and a power of 80% (type II error = 0.20).

### Recruitment and randomization

From April 2017 to May 2018, as part of the triage evaluation, all patients accessing the ED were asked about their smoking habits. Smokers were then proposed to participate in the study. Once the input of the clinical data and the oral consent were obtained from the patients, these latter were randomly allocated to the control or intervention arm by the hospital management software and were given the clinical documents needed for the visit together with the study informed consent sheet. A printed univocal mark at the bottom of this informed consent allowed the healthcare providers to identify those patients enrolled in the study according to their allocation. Enrolled subjects were blinded to the assigned arm. Patients who gave written information consent were also asked to fill out a questionnaire aimed to investigate the characteristics of their smoking habits.

A dedicated operator, not part of the ED staff, took care of all aspects pertaining to the collection of the informed consents and questionnaires and acted as liaison between the nurses and the patients in the intervention group.

### Intervention

**Figure 1:**
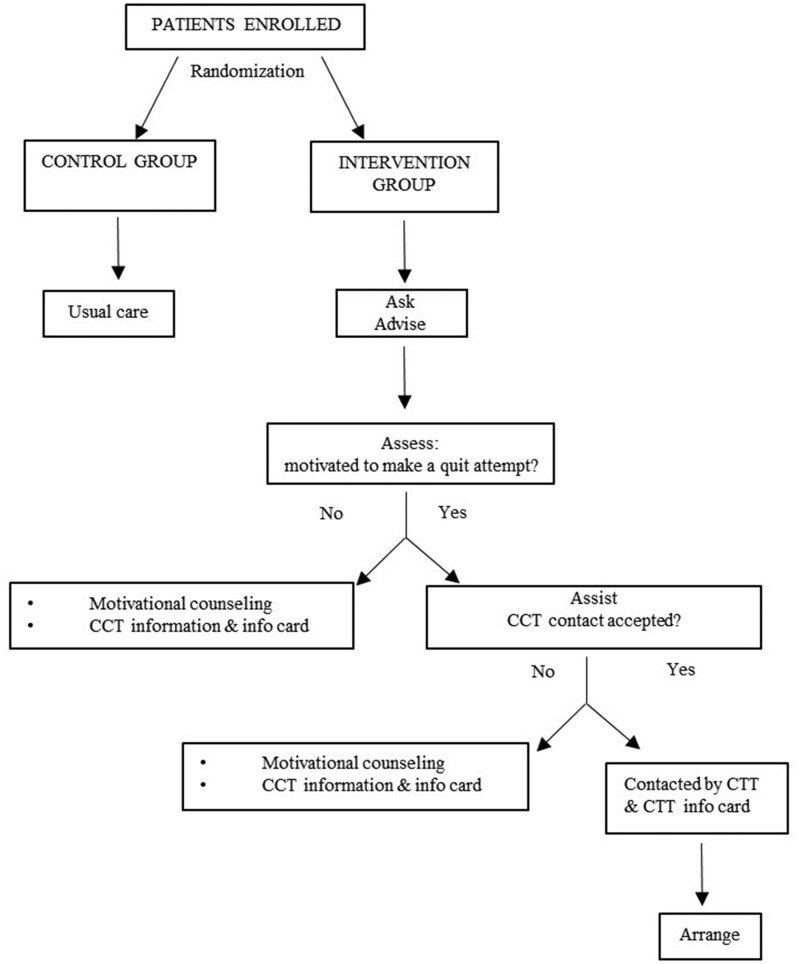
Intervention flowchart. CTT: Centro per il Trattamento del Tabagismo (Italian for “Smoking Cessation Center”)

The intervention group received a brief counselling based on a 5A’s model (figure 1) partly adapted to the study context.^13^^,^^14^ Specifically, at step 4 (i.e. Assist), patients motivated to quit were asked whether they would agree to be contacted by the *Centro per il Trattamento del Tabagismo* (CTT) (Italian for ‘Smoking Cessation Center’), inside the Department of Addiction of the Novara’s Local Health Unity in order to plan intensive counselling. Instead, patients not motivated to quit were only administered motivational counselling.

All patients of the intervention group received a card containing information on the CTT, a few dedicated website links and the telephone number and location of the CTT. Motivated patients were referred to the CTT, and, within a few days from the ED visit, these patients were contacted by a CTT operator in order to schedule their first meeting.

The brief intervention was carried out by the nurse who took clinical care of the patient. All the nurses acting as ‘intervention providers’ received specific training from the CTT staff. This training was specifically focused on the description and application of the 5A’s intervention, the importance of the relationship with patients and the acquisition of specific skills through role play techniques. The training session was conducted a few months before the beginning of the enrolment and consisted of 2 h of frontal lesson and 2 h of role-playing exercises. No re-training courses were scheduled.

Control patients received the ‘usual care’, which consisted in advising them to quit without conducting structured counselling.

The actual implementation of every step of the ‘intervention as expected’ was recorded in a process checklist. It included information on the implementation of tasks and the reasons for lack of implementation. Finally, at the end of the recruitment phase, an anonymous survey was administered to the nurses involved in the study to address barriers to implementing 5A’s counselling.

### Outcomes

The primary outcome was the point prevalence of tobacco-free patients (i.e. those who did not smoke at the moment of the follow-up call) both at 6 and 12 months from the intervention.

The secondary outcomes were (i) consecutive past 30-day smoking abstinence at 6- and 12-month follow-up; (ii) past 7-day 50%, or more, decrease in daily tobacco consumption over baseline at 6- and 12-month follow-up; and (iii) the number of attempts to quit smoking at 6- and 12-month follow-up. All the information was self-reported.

### Follow-up

The follow-up was performed at 6 and 12 months through computer-assisted telephone interview. In order to minimize dropouts, three call attempts were made before considering the patient as lost to follow-up.

### Data management and statistical analysis

Demographic and clinical characteristics of treatment and control groups were compared to assess the balance across the experimental arms at baseline using chi-square for categorical variables or *t*-tests for continuous variables. Absolute (*N*) and percentage (%) frequencies were reported for the categorical variables, while mean and standard deviation were reported for the numeric ones.

The outcomes were dichotomized, and RRs for the expected outcomes, with their 95% confidence intervals (CIs), were obtained by comparing the experimental with the control group. The effects of the treatments were evaluated using intention-to-treat (ITT) analyses. A sensitivity analysis using the per protocol (PP) approach was performed to evaluate the possible role of the intervention among adherent patients.

All data were recorded through RedCap^®^ software, thereby ensuring confidentiality and high privacy level according to the Italian law. All statistical analyses were performed using SAS software (Copyright © 2013, SAS Institute Inc., Cary, NC, USA).

### Process research survey

Given the low level of adherence to the study procedures, a questionnaire addressing barriers to the implementation of the intervention was prepared and administered to all nurses involved in the study. This was later analysed in a descriptive way. For numeric variables, median and interquartile ranges (IQR) were used.

## Results

From April 2017 to May 2018, 890 smokers admitted to the ED of the Novara University Hospital were asked to participate in the study. Among these patients, 293 (32.9%) refused to participate, while 597 (67.1%) gave their verbal informed consent and were thus randomized. However, of these latter, only 480 subjects (80.4%) filled out the baseline questionnaire and were thus included in the study, which comprised 262 subjects randomized to the intervention group and 218 subjects allocated to the control group.

**Figure 2:**
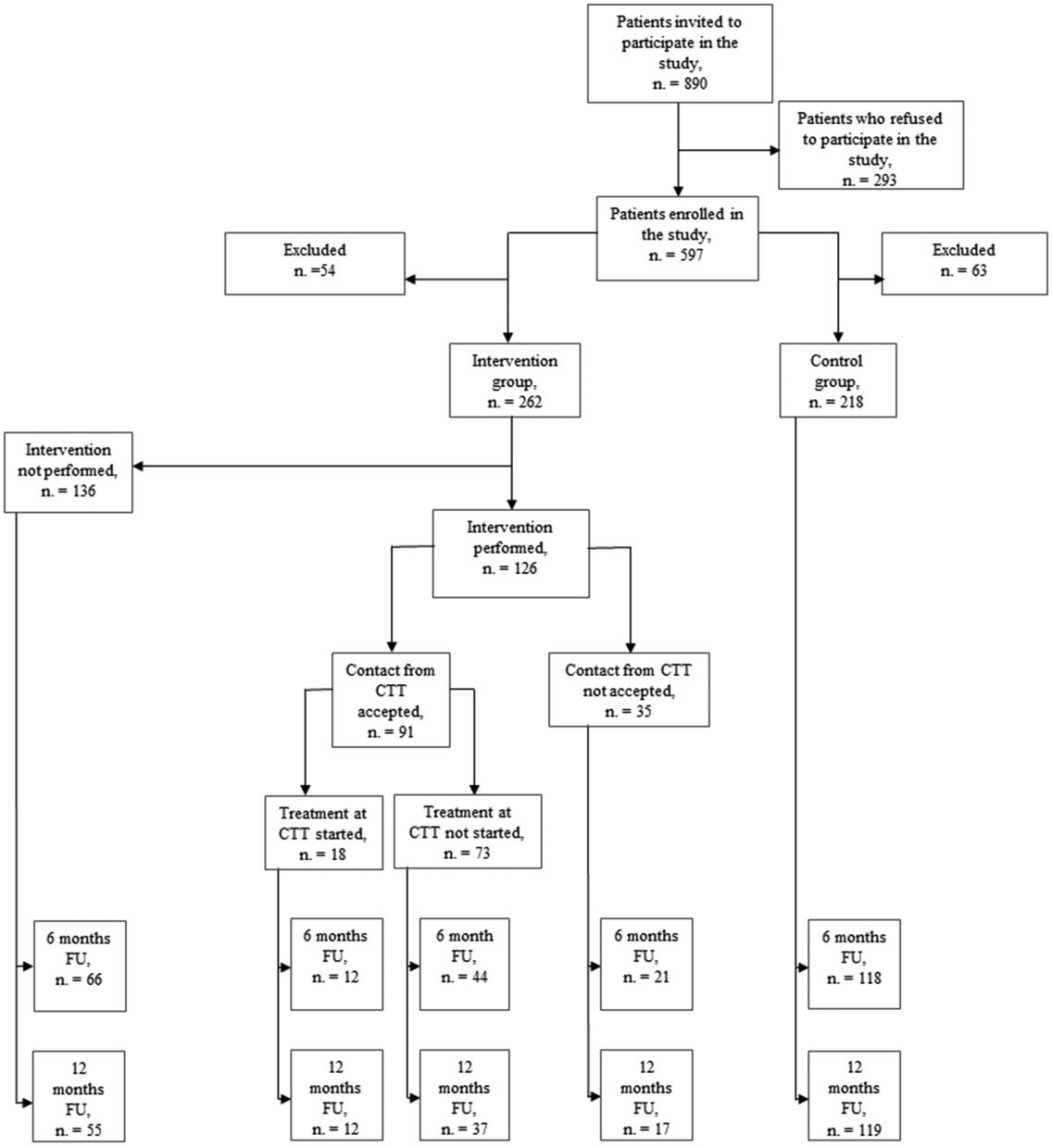
Patient enrollment and exclusion. CTT: Centro per il Trattamento del Tabagismo (Italian for “Smoking Cessation Center”); FU: follow-up

In the intervention group, 126 out of 262 subjects (48.1%) received smoking cessation counselling, with 91 of them (72.2%) accepting to be contacted by the CTT and 18 starting a treatment at the CTT (figure 2).

### Baseline characteristics

The patients enrolled in the study were predominantly males (*N* = 295, 61.5%), with a mean age of 43.7 ± 14.8 years. The most frequently assigned priority code at triage was green (*n* = 391, 81.5%). Furthermore, only a limited number of patients were admitted to the ED for a potential smoking-related condition: 16.8% (*n* = 44) in the intervention group and 17.4% (*n* = 38) in the control group. The mean daily number of cigarettes smoked did not vary between the two study groups, with most of the patients showing a low-to-moderate dependence according to the Fagerström test score.^19^ As listed in table 1, no significant differences between the main baseline characteristics of the two groups were observed.

**Table 1:** Baseline patient characteristics

Patients characteristics	Total (*n* = 480), *n* (%)/mean ± SD	Intervention group (*n* = 262), *n* (%)/mean ± SD	Control group (*n* = 218), *n* (%)/mean ± SD	*P*-value
Age	43.7 ± 14.8	44.7 ± 14.8	42.5 ± 14.8	0.101
Males	295 (61.5%)	164 (62.6%)	131 (60.1%)	0.575
Smoking-related conditions	82 (17.1%)	44 (16.8%)	38 (17.4%)	0.950
Priority code				
White	16 (3.3%)	10 (3.8%)	6 (2.7%)	
Green	391 (81.5%)	211 (80.5%)	180 (82.6%)	0.7634
Yellow	73 (15.2%)	41 (15.6%)	32 (14.7%)	
Cigarettes per day	14.40 ± 8.3	14.50 ± 8.5	14.20 ± 8.1	0.690
Years of smoking	21.97 ± 14.7	22.30 ± 14.6	21.90 ± 14.9	0.730
Previous quit attempts	342 (71.2%)	188 (72.0%)	154 (71.3%)	0.940
Willingness to make a quit attempt	63/132 (47.7%)	37/71 (52.1%)	26/61 (42.6%)	0.550
Willingness to make further quit attempts	245/327 (74.9%)	137/179 (76.5%)	108/148 (73.0%)	0.521
Fagerström test score				
0–2 = low dependence	173/470 (36.8%)	100/258 (38.8%)	73/212 (34.4%)	
3–4 = moderate dependence	150/470 (31.9%)	79/258 (30.6%)	71/212 (33.5%)	0.490
5–6 = high dependence	106/470 (22.6%)	57/258 (22.1%)	49/212 (23.1%)	
7–10 = very high dependence	41/470 (8.70%)	22/258 (8.5%)	19/212 (9.0%)	
Chronic diseases				
Diabetes	26/469 (5.5%)	17/257 (6.6%)	9/212 (4.2%)	0.361
COPD	14/470 (3.0%)	5/257 (1.9%)	9/213 (4.2%)	0.240
Ischaemic heart disease	16/470 (3.4%)	10/257 (3.9%)	6/213 (2.8%)	0.701
Stroke	6/470 (1.3%)	5/257 (1.9%)	1/213 (0.5%)	0.314
Cancer	13/470 (2.8%)	5/257 (1.9%)	8/213 (3.8%)	0.363
Depression	34/469 (72.5%)	19/256 (7.4%)	15/213 (7.0%)	0.983
Other chronic diseases	150/449 (33.4%)	86/245 (35.1%)	64/204 (31.40%)	0.463

Categorical variables are reported as absolute numbers and percentages. Numerical variables are reported as mean ± standard deviation (SD). Categorical variables were compared using the chi-square test, while numerical variables were analysed by paired *t*-test. Statistical significance was set at *P *<* *0.05.

COPD, chronic obstructive pulmonary disease.

### Outcome results

ITT analysis did not detect any differences in primary and secondary outcomes between the intervention and control group (table 2). At 6-month follow-up, a slight but not statistically significant increase in smoking cessation was observed in the intervention group (RR = 1.04, 95% CI = 0.58–1.87), while an opposite observation was made at 12-month follow-up (RR = 0.86, 95% CI = 0.50–1.47). Similar results were obtained for the secondary outcomes.

**Table 2:** Primary and secondary outcome results at 6- and 12-month follow-up according to ITT and PP analysis

ITT analysis						
Outcomes	6-Month follow-up			12-Month follow-up		
	Intervention group (*n* = 143)	Control group (*n* = 118)	RR (CI 95%)	Intervention group (*n* = 121)	Control group (*n* = 119)	RR (CI 95%)
Primary outcome						
Tobacco-free patients	22 (15.4%)	17 (14.8%)	1.04 (0.58–1.87)	20 (16.5%)	23 (19.33%)	0.86 (0.50–1.47)
Secondary outcomes						
Continuous abstinence during the last 30 days	19 (13.4%)	14 (12.2%)	1.10 (0.58–2.10)	18 (14.9%)	18 (15.13%)	0.98 (0.54–1.18)
Decrease of at least 50% in daily tobacco use	38 (27.3%)	28 (25.5%)	1.07 (0.71–1.63)	41 (34.5%)	40 (35.09%)	0.98 (0.69–1.40)
At least one quit attempt	55 (38.7%)	47 (41.6%)	0.93 (0.69–1.26)	52 (43.0%)	50 (42.02%)	1.02 (0.76–1.37)

**PP analysis**						
**Outcomes**	**6-Month follow-up**			**12-Month follow-up**		
	**Intervention group (*n* = 77)**	**Control group (*n* = 118)**	**RR (CI 95%)**	**Intervention group (*n* = 66)**	**Control group (*n* = 119)**	**RR (CI 95%)**

Primary outcome						
Tobacco-free patients	14 (18.2%)	17 (14.8%)	1.23 (0.64–2.35)	14 (21.2%)	23 (19.3%)	1.10 (0.61–1.98)
Secondary outcomes						
Continuous abstinence during the last 30 days	11 (14.5%)	14 (12.2%)	1.19 (0.57–2.48)	13 (19.7%)	18 (15.1%)	1.30 (0.60–2.49)
Decrease of at least 50% in daily tobacco use	22 (29.7%)	278 (25.5%)	1.17 (0.73–1.88)	24 (37.5%)	40 (35.1%)	1.07 (0.71–1.60)
At least one quit attempt	33 (42.9%)	47 (38.8%)	1.11 (0.81–1.52)	33 (50.0%)	50 (42.0%)	1.19 (0.86–1.64)

CI, confidence interval; ITT, intention to treat; PP, per protocol; RR, relative risk.

When we performed PP analysis of patients adhering to the protocol (table 2), we found that the likelihood of quitting smoking showed an upward trend, albeit not statistically significant, in the intervention group at both 6 (RR = 1.23, 95% CI = 0.64–2.35) and 12 months (RR = 1.10, 95% CI = 0.61–1.98) compared to the control group. Similarly, the RR value of the other outcomes ranged from 1.14 to 1.23 at 6 months and from 1.07 to 1.19 at 12 months.

Ninety-one patients of the intervention group accepted to be contacted by the CTT staff, in order to schedule the first visit. Only 61 (67.0%) of them were actually contacted and 37 accepted to present themselves at the CTT to start a treatment. The other 24 declared to be not sufficiently motivated or not interested, or to have already quit. Among those having accepted, however, only 18 subjects actually attended the first visit and started the treatment program. Notably, 6 of these subjects were loss to follow-up, while the remaining 12 (66.7%) completed the treatment and were smoke-free—according to hair analysis and expiratory Carbon moxodine tests—at subsequent follow-ups. 

### Intervention process data

The data relative to the intervention process, available for 101 out of 136 patients randomized to the intervention group who not received the smoking cessation counselling, provided an explanation for the non-adherence. While in 41.9% of cases counselling had not been administered due to organizational issues, such as time constraint, 34.6% of patients had refused the treatment. In the remainder of cases the survey had not been filled out.

### Post-study survey results

All 37 nurses originally trained for the study were invited to participate in a post-study survey, but only 19 (51.4%) of them completed it. The responders estimated to have screened a median of 50 subjects (IQR 18–130), to have enrolled a median of 6 patients (IQR 5–14) and to have performed a median of 5 interventions (IQR 5–20) during the whole study period.

The median self-reported feasibility level of the complete study procedure—from enrolment to treatment—was 38 (IQR 18–59). The main issues reported were time restrictions (73.7%), high refusal rate after screening (68.4%) and high number of patients withdrawing their consent (11%).

Overall, even though nurses regarded 5A’s counselling as being important (median 50 with IQR 30–74), they considered such intervention as not feasible in the ED setting (median 31, IQR 18–50). Among those answering the questionnaire, five nurses (26.3%) were current smokers, whereas the majority of them stated that individual smoking habits did not affect the way they counselled patients.

## Discussion

This RCT investigated the feasibility and effectiveness of 5A’s brief and structured counselling administered by trained nurses, an intervention aimed to promote smoking cessation in Italian smokers admitted to the ED.

The ITT analysis did not detect any differences in primary and secondary outcomes between intervention and control group, both at 6- and at 12-month follow-up. Given the low rate of adherences to the protocol (<50%), a PP analysis was also carried out, revealing a slightly higher prevalence of tobacco-free patients in the intervention group at 6-month follow-up, compared to the control group, a trend that decreased at 12 months. These results are consistent with two previous studies performed in the ED setting,^12^^,^^13^ showing a promising but weak positive trend in the outcome of patients in the intervention vs. control group. Importantly, we show that of the 18 patients who were actually referred to and treated at our CTT, 12 completed the smoking cessation therapy and were smoke-free at the second follow-up.

With regard to feasibility of 5A’s counselling, our findings suggest that such intervention should not be recommended in the ED setting for the following two main reasons:

- Only 126 out of 316 smokers randomized to the intervention group were actually given counselling. Furthermore, despite this very low number of candidates, the nurses were able to counsel only 40% of them. In the vast majority of cases, the nurses identified ‘lack of time’ as the main reason for not being able to administer the counselling. The unfeasibility of such intervention can therefore be ascribed to current working conditions of healthcare professionals in understaffed and overcrowded public Italian hospitals, especially EDs. Thus, in light of these findings, future efforts in promoting smoking cessation, rather than creating additional workload for ED staff, should target a wider audience by means of informative posters and brochures displayed in the ED waiting room, promoting smoking cessation and referring patients to local CTTs.
- The observation that 33.3% of smokers admitted to the ED refused to participate in the intervention and 11.7% did not fill out the questionnaire, together with the fact that only 12 out of 126 smokers referred to the local CTT actually completed their treatment, indicates that the ED stay is not a teachable moment for smokers, at least in this Italian setting. Indeed, it is conceivable that their high level of stress due to an overcrowded environment combined their heightened psychological distress can make ED patients less receptive to prevention messages.^20^

Previous experiences in different health settings showed better results than our study; e.g. the FRICC study that was conducted in dental clinics.^21^ The strengths of such approach were the large number of patients requiring dental care and the high association between oral and dental diseases and smoking. Other studies evaluated primary care providers^22^^,^^23^ and family physician.^24^ The so called ‘Ottawa Model’,^25^ was based on a systematic intervention provided to all the hospitalized smokers in nine hospitals in Ontario; the model allowed to significantly increase the confirmed 6-month continuous abstinence rate. Other successful studies were focused on specific group of patients, mainly affected by tobacco related cancers.^26^^,^^27^ This latter approach, in our opinion, is completely different from ours, since it aims to a narrower target of patients and its goal is tertiary prevention.

Taken together, these observations lead us to understand that, in the future, any smoking cessation program should be developed in other clinical contexts rather than in an ED similar to our one: previous experiences showed that high accession facilities represent an optimal setting for tobacco cessation interventions but, in our opinion, our ED mismatched two major requirements: a quiet environment and enough time availability.

A limitation of this study is represented by the limited size of our study population. Even though the period of enrolment was extended from 5 to 14 months to reach the expected number of participants (*n* = 1200), only 816 subjects were screened for inclusion. In these 30 months, an estimated number of 20 000 smokers admitted to the ED of Novara could have been potentially included in the study. Thus, given the low statistical power of our study, we cannot definitively rule out that the smoking cessation counselling given to ED patients may have proven effective had we analysed a larger population.

Another limitation is represented by the sub-optimal screening and enrolment procedures. While these tasks should have been carried out by specifically trained ED personnel throughout the course of the study, most of them were concentrated in a few months and conducted by an *ad* *hoc* recruited junior researcher.

In conclusion, a well-standardized intervention conducted by trained personnel in the framework of an RCT coordinated by a locally renowned university shows an extremely and disappointing low level of implementation. Moreover, the relatively low number of smokers enrolled, accompanied by a high number of non-adherents and dropouts occurring during all the phases of the study, from enrolment to adherence to the treatment and to attendance to the follow-up implies that the ED stay should not be considered as a teachable moment. Nevertheless, the high rate of smoke-free patients among those receiving the treatment in the CTT—12 out of 18 patients at the 6-month follow-up suggest that a close cooperation between CTTs and EDs may favour smoking cessation in those patients who are motivated to quit. Thus, we strongly believe that efforts should be undertaken to implement a ‘fast track’ smoking cessation plan allowing ED physicians to promptly refer ready-to-quit smoker patients to a CTT.
